# Radioprotective effects of oral 17-dimethylaminoethylamino-17-demethoxygeldanamycin in mice: bone marrow and small intestine

**DOI:** 10.1186/2045-3701-3-36

**Published:** 2013-09-16

**Authors:** Xinyue Lu, Dilber Nurmemet, David L Bolduc, Thomas B Elliott, Juliann G Kiang

**Affiliations:** 1 Radiation Combined Injury Program, Scientific Research Department, Armed Forces Radiobiology Research Institute, 8901 Wisconsin Avenue, Bethesda, MD 20889-5603, USA; 2 Department of Radiation Biology, Uniformed Services University of the Health Sciences, Bethesda, MD, USA; 3 Department of Medicine, Uniformed Services University of the Health Sciences, Bethesda, MD, USA

**Keywords:** Radiation, Survival, 17-DMAG, Bone marrow, Stem cell, Intestine, Lgr5, Survivin, G-CSF, Mice

## Abstract

**Background:**

Our previous research demonstrated that one subcutaneous injection of 17-Dimethylaminoethylamino-17-demethoxygeldanamycin (17-DMAG) 24 hours (h) before irradiation (8.75 Gy) increased mouse survival by 75%. However, the protective mechanism of 17-DMAG is currently unknown. The present study aimed to investigate whether oral administration of 17-DMAG was also radioprotective and the potential role it may play in radioprotection.

**Results:**

A single dose of orally pre-administered (24, 48, or 72 h) 17-DMAG (10 mg/kg) increased irradiated mouse survival, reduced body weight loss, improved water consumption, and decreased facial dropsy, whereas orally post-administered 17-DMAG failed. Additional oral doses of pre-treatment did not improve 30-day survival. The protective effect of multiple pre-administrations (2−3 times) of 17-DMAG at 10 mg/kg was equal to the outcome of a single pre-treatment. In 17-DMAG-pretreated mice, attenuation of bone marrow aplasia in femurs 30 days after irradiation with recovered expressions of cluster of differentiation 34, 44 (CD34, CD44), and survivin in bone marrow cells were observed. 17-DMAG also elevated serum granulocyte-colony stimulating factor (G-CSF), decreased serum fms-related tyrosine kinase 3 ligand, and reduced white blood cell depletion. 17-DMAG ameliorated small intestinal histological damage, promoted recovery of villus heights and intestinal crypts including stem cells, where increased leucine-rich repeat-containing G-protein coupled receptor 5 (Lgr5) was found 30 days after irradiation.

**Conclusions:**

17-DMAG is a potential radioprotectant for bone marrow and small intestine that results in survival improvement.

## Background

17-dimethylamino-ethylamino-17-demethoxygeldanamycin (17-DMAG) was a derivative of heat shock protein 90 (Hsp90) inhibitor geldanamycin [1,2]. Compared to geldanamycin, 17-DMAG was greater water-soluble and lower hepatotoxic [3], and was distributed more rapidly in tissues. These advantages led to superior clinical applications in human tumor therapy [4,5]. Human cancer studies [6,7] showed the mean half-life of 17-DMAG to be 22.3-24 h, which was much longer than what has been previously observed in mice (69–88 min) after an intravenous (i.v.) injection [8]. The drug was well tolerated over periods of months of use even when combined with other medical treatments such as radiation therapy [4,9]. *In vitro*, 17-DMAG selectively killed human tumor cells by chelating Hsp90 in its high-affinity conformation, which existed only in cancer cells [10], and thereby prevented mutant proteins from entering the nucleus to trigger gene activation [11]. Because of the roles that Hsp90 played in key processes of tumor growth and development, including induction and stabilization of growth factors and other signals in transformed cells, angiogenesis, and promotion of metastasis, Hsp90 understandably became an inviting target in the search for pharmaceutical agents to kill cancer cells.

Recent reports from our laboratory revealed that 17-DMAG protected human T-cells and normal healthy peripheral blood mononuclear cells from γ-photon radiation *in vitro and ex vivo*[12,13]. The protective effects were associated with inhibiting inducible nitric oxide synthase (iNOS)/caspase-3 cascade [12] and protein 53 (p53) accumulations [13] to prevent apoptosis in irradiated cells. Those results imply that Hsp90 inhibitors such as 17-DMAG could be useful not only for cancer therapy, but also for general radioprotection for normal healthy tissues. 17-DMAG was, therefore, considered as a candidate radioprotectant for total-body irradiation countermeasure *in vivo*.

Total-body exposure to ionizing radiation in humans and animals results in multiple organ injury and dysfunction. Ionizing radiation injured hematopoietic, gastrointestinal, or cerebrovascular systems depend on the total dose of radiation received [14,15]. Safe and effective radioprotectors were needed in the event of a radiological warfare or accident, a nuclear terrorist attack, radiation therapy, or prolonged space travel [14-18].

Our previous data demonstrated the potential of 17-DMAG as a radioprotector. A subcutaneous (s.c.) injection of a single dose of 17-DMAG (25 mg/kg) given to CD2F1 mice 24 h before a lethal dose of gamma-radiation at 8.75 Gy (i.e., LD_70/30,_ a dose resulting in 70% of exposed population died within 30 days in a study [19]) improved mouse 30-day survival by 75 percentage points with a dose reduction factor (DRF) of 1.2 [20]. However, oral administration of prophylactic or therapeutic drugs is a most desirable and feasible remedy of prevention or treatment in civilian and military mass radiation casualties from a radiation accident, or fist responders entering radiation contaminated areas. It is, therefore, of great interest to determine whether orally administered 17-DMAG was also radioprotective for survival, bone marrow, and small intestine. We hypothesized that 17-DMAG given orally would provide radioprotection but, possibly, at various levels of effectiveness in comparison to s.c. administration. In this study, we investigated the radioprotective efficacy and possible underlying mechanisms of 17-DMAG when it was administered orally in mice prior to ionizing irradiation. This is the first report that orally administered 17-DMAG provided prophylactic reduction of ionizing radiation-induced lethality, bone marrow damage, and small intestine injury in CD2F1 mice. This radioprotective activity was demonstrated in (1) partially preventing bone marrow cell damage with the consequence of upregulated granulocyte-colony stimulating factor (G-CSF) in serum, decreased fms-like tyrosine kinase-3 (Flt-3) ligand in serum, and increased survivin expression in bone marrow cells, which may further promote bone marrow recovery, and (2) ameliorating the radiation-induced injury in small intestinal crypt cells perhaps by increased leucine-rich repeat-containing G-protein coupled receptor 5 (Lgr5)-positive crypt cells, which may accelerate villus repairing and recovery 30 days post-irradiation.

## Results

### Orally administered 17-DMAG prior to irradiation increased mouse survival

**Figure 1:**
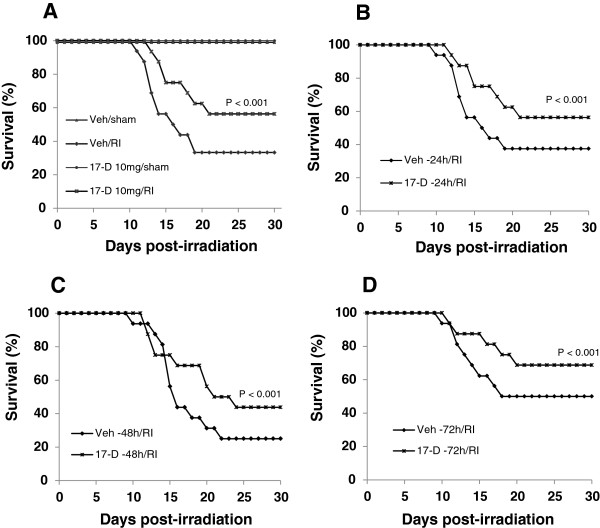
**Orally administered 17-DMAG prior to irradiation increased mouse survival. (A)** Administration of a single oral dose of 17-DMAG (10 mg/kg) 24 h prior to irradiation at 8.75 Gy increased mouse 30-day survival by 20–25 % (p < 0.001, n = 16 mice per group) versus (vs.) vehicle-treated irradiated (Veh/RI) animals. Furthermore, pretreatment of a single oral dose of 17-DMAG given 24 to 72 h before irradiation at 8.75 Gy was still effective to improve survival**.** 17-DMAG (10 mg/kg) given 24 **(B)**, 48 **(C)**, or 72 h **(D)** before irradiation increased survival of irradiated mice by 20% above the vehicle-treated irradiated mice (p < 0.001, n = 16 per group). Veh: vehicle; 17-D: 17-DMAG; RI: radiation injury.

Mice received a single oral dose of 5, 10, 25, or 75 mg/kg 17-DMAG 24 h before irradiation at 8.75 Gy (i.e. LD_75/30_). In vehicle-treated mice, radiation-induced mortality started at day 9 after irradiation and the 30-day survival was 33.5% (Figure 1A). A single oral dose of 10 mg/kg 17-DMAG protected 56.3% of mice from radiation-induced mortality. The onset of mortality began at 11.6 d, 2.5 days later than the vehicle-treated mice (11.625 ± 0.296 vs. 9.125 ± 0.5625, p < 0.05, n = 4 repeated experiments) after irradiation and prolonged the time to reach 50% survival (ST_50_) approximately by 8 days (≥25.25 ± 2.75 vs.17.00 ± 0.89, p < 0.05, n = 4 repeated experiments). The dose-modifying factor (DMF) [18] for oral administration of 17-DMAG 24 h before irradiation (ratio of survival of animals treated with 17-DMAG to survival of the untreated animals) was 56.3/33.5 = 1.68. However, pretreatment with oral doses of 5, 25, and 75 mg/kg did not improve mouse 30-day survival after irradiation (data not shown), suggesting a narrow effective dose range via oral route in irradiated CD2F1 mouse. 17-DMAG administered alone up to 75 mg/kg without irradiation did not cause mortality in sham animals (data not shown).

### 17-DMAG induced radioprotection for up to three days before irradiation

The optimal oral administration time was determined at the dose of 10 mg/kg of 17-DMAG as shown in Figure 1A-D, oral administration of 17-DMAG 24, 48, or 72 h before irradiation appeared to increase mouse survival 20–25 percentage points above the corresponding vehicle-treated group (p < 0.001). When the same dose of 17-DMAG was given orally −1 h, however, no any survival improvement was found.

### Multiple oral administrations of 17-DMAG induced the same radioprotective efficacy as a single administration

**Figure 2:**
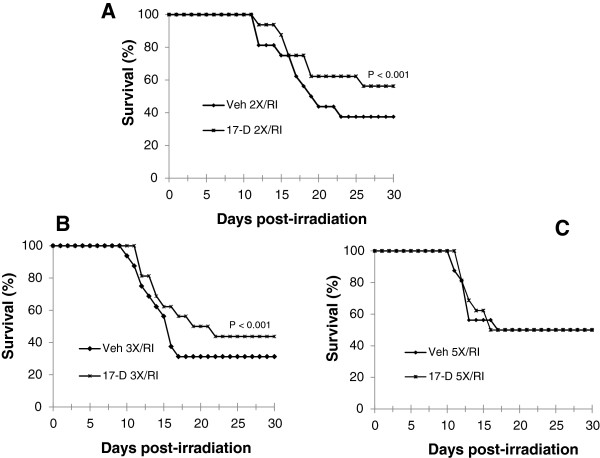
**Multiple oral administrations of 17-DMAG induced a similar radioprotective efficacy to a single administration. (A)** 17-DMAG (10 mg/kg) or vehicle was given twice (2×) at −48 and −24 h, **(B)** given three times (3×) at −72, −48, and −24 h, or **(C)** given five times (5×) at −120, −96, −72, −48, and −24 h prior to irradiation at 8.75 Gy (N = 16 mice per group). Veh: vehicle; 17-D: 17-DMAG; RI: radiation injury.

Multiple oral administrations with 17-DMAG did not further increase its radioprotective efficacy (Figure 2). The percentage of survival in mice given a dose of 10 mg/kg 17-DMAG 2 or 3 times before irradiation was similar to the survival percentage observed in mice treated with a single dose of 17-DMAG (compared to Figure 1B). However, 17-DMAG given 5 times did not improve survival compared with irradiated animals pretreated with 5 times of vehicle.

### Oral administration of 17-DMAG after irradiation did not increase survival

To determine if a single oral dose of 17-DMAG was capable of producing therapeutic efficacy, mice received 10 mg/kg 17-DMAG orally, 1, 6, or 24 h after irradiation. All oral administrations of 17-DMAG post-irradiation failed to improve survival (data not shown).

### 17-DMAG attenuated body weight loss, promoted recovery of reduced water consumption, and decreased facial dropsy after irradiation

**Figure 3:**
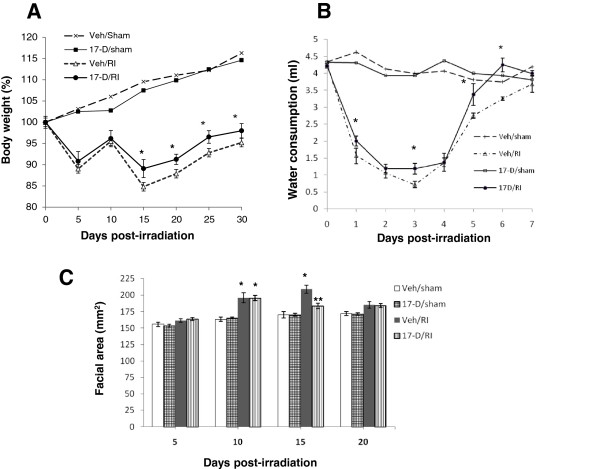
**17-DMAG attenuated body weight loss and promoted recovery of reduced water consumption as well as facial dropsy.** Mice were orally administered with 10 mg/kg 17-DMAG or vehicle 24 h prior to irradiation at 8.75 Gy (N = 16 mice per group). Their body weights and facial dropsy were monitored for 30 days. Water consumption was measured daily for the first 7 days. **(A)** Attenuation of body-weight loss during days 15 to 30 after irradiation, * p = 0.03 vs. Veh/RI. Error bars indicate the standard error of the mean (SEM) for 5−16 mice per group at different days. **(B)** Improvement of water consumption at days 1, 3, 5, and 6 during the first 7 days after irradiation, * p = 0.03 vs. Veh/RI group. Error bars indicate the standard error of the SEM for 16 mice per group at different days. **(C)** Murine facial area at days 5, 10, 15, and 20 after irradiation. At day 10, * p < 0.01 vs. Veh/sham and 17-D/sham, and error bars indicate the SEM for 13−16 mice per group; at day 15, * p < 0.01 vs. Veh/sham, 17-D/sham, and 17-D/RI; ** p < 0.001 vs. Veh/RI, error bars indicate the SEM for 9−16 mice per group. Veh: vehicle; 17-D: 17-DMAG; RI: radiation injury.

Based on the above results, mice received a single dose of 10 mg/kg 17-DMAG 24 h prior to irradiation in the following experiments to investigate changes in body weight, water consumption, and facial dropsy. Irradiation reduced body weights and water consumption [21], but did not induce facial edema in B6D2F1/J mice. As shown in Figure 3A, in CD2F1 mice, irradiation began to reduce the body weight at day 5 and remained low. Irradiation made mice consume less water from day 1 through day 5. On day 6 the mice increased their water intake that returned to the baseline on day 7 (Figure 3B). Irradiation insult also induced facial dropsy, which began to occur approximately at day 10 (Figure 3C) and seemed to be one of animal moribund signs in this strain. Compared to vehicle group, 17-DMAG administration significantly prevented the radiation-induced loss of body weights during days 15 to 30 (Figure 3A), ameliorated a reduction of water consumption at days 1, 3, 5, and 6 (Figure 3B). It may be inferred that administration of this drug provided some degree of protection from gastrointestinal injury or dysfunction after irradiation. In addition, an attenuation of facial dropsy was observed at day 15 postirradiation in animals pretreated with 17-DMAG. At day 20 after irradiation, no significant differences of facial dropsy were observed between vehicle- and 17-DMAG-treated surviving mice (Figure 3C).

### 17-DMAG attenuated bone marrow damage after irradiation

Mice that received a single oral dose of 10 mg/kg 17-DMAG 24 h prior to irradiation were also used to investigate selected cellular and molecular changes in order to elucidate the protective mechanism.

**Figure 4:**
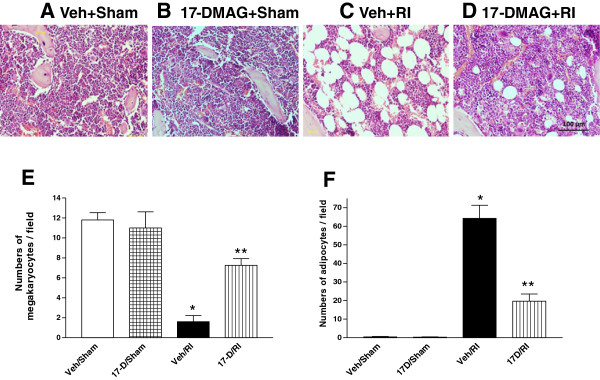
**17-DMAG attenuated bone marrow damage after irradiation.** Morphological alteration in femur bone marrows of mice pretreated with **(A)** vehicle + sham, **(B)** 17-DMAG + sham, **(C)** vehicle + RI, and **(D)** 17-DMAG + RI 30 days after irradiation at 8.75 Gy. The images were from epiphyseal ends with H & E staining, 20 × magnification. A 100 μm scale bar was shown on the bottom of right panel D. Radiation induced vast reduction of hematopoietic cellularity and replacement by adipocytes in bone marrow. However, **(E)** a significantly improved number of megakaryocytes and **(F)** a significantly decreased numbers of adipocytes were found per field under microscopy (20 ×) in bone marrows of 17-DMAG-pretreated mice compared to those in vehicle-pretreated group 30 days after irradiation. * p < 0.001 vs. Veh/sham, 17-D/sham, and 17-D/RI; ** p < 0.01 vs. Veh/Sham, 17-D/sham, and Veh/RI group. Error bars indicate the SEM for at least 4–5 independent experiments. Veh: vehicle; 17-D: 17-DMAG; RI: radiation injury.

Bone marrow was examined for its morphological and molecular alterations in surviving mice on the 30th day postirradiation. Pathological changes in sections of femoral bone marrows showed that irradiation reduced marrow cellularity, thereby resulting in the absence of erythroid, rare myeloid and megakaryocytic cells. Many fat vacuoles appeared when stem cells and their lineages, especial megakaryocytes were depleted in the bone marrow (Figure 4C vs. A and B). In contrast, microfoci were regenerated partially (Figure 4D vs. C), megakaryocytes were restored significantly (Figure 4E), and adipogenesis was reduced after 17-DMAG administration (Figure 4F).

**Figure 5:**
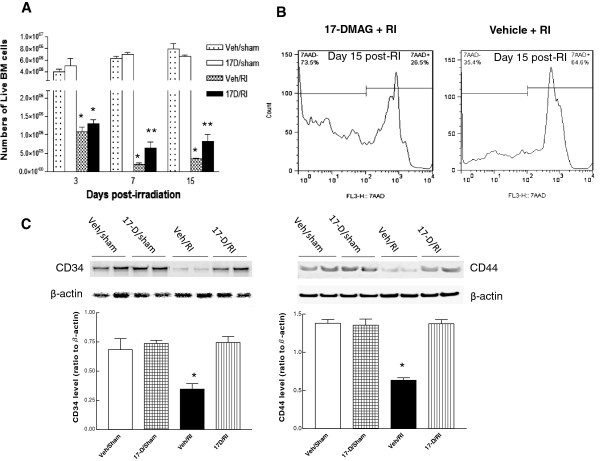
**17-DMAG significantly promoted bone marrow cell viability after irradiation.** Mice were administered with 17-DMAG or vehicle 24 h prior to irradiation at 9.25 Gy. **(A)** Bone marrow cell viability by trypan blue exclusion: total numbers of live bone marrow cells collected from each femur bone of mouse at days 3, 7 or 15 after irradiation were measured by trypan blue staining. Irradiation reduced bone marrow cell viability with a significant reduction of total live cell numbers in all three time points. 17-DMAG significantly increased bone marrow cell viability at days 7 and 15 after irradiation (N = 4–6 mice per group). At day 3, * p < 0.05 vs. Veh/sham, 17D/sham. At days 7 and 15, * p < 0.05 vs. Veh/sham, 17D/sham, and 17D/RI; ** p < 0.05 vs. Veh/sham, 17D/sham, and Veh/RI. Error bars indicate the SEM for 4–6 independent experiments at various time points. **(B)** Bone marrow cell viability by flow cytometric assay: twenty thousands of bone marrow cells from each femur bone of mouse pre-treated with either 17-DMAG (left) or vehicle (right) at day 15 after irradiation were stained with 7-aminoactinomycin D (7AAD). 17-DMAG increased bone marrow cell viability with a greater percentage of 7AAD-negative cells. Error bars indicate the SEM for at least three independent experiments. **(C)** Using Western blotting, decreased CD34 and CD 44 protein were detected in bone marrow cells from femur bone of Veh/RI-mice at day 30 after irradiation at 8.75 Gy; 17-DMAG blocked the RI-induced suppression in both CD34 and 44 protein expression at day 30 after irradiation. * p < 0.05 vs. Veh/sham, 17-D/sham, and 17D/RI. Error bars indicate the SEM for 4–5 independent experiments. Veh: vehicle; 17-D: 17-DMAG; RI: radiation injury.

A separate experiment was performed in order to determine the effects of 17-DMAG on the survival of bone marrow cells in irradiated mice. Bone marrow cells were collected from femora at days 3, 7 and 15 after irradiation and total live bone marrow cells from each murine femora were determined by trypan blue staining. Gamma-irradiation induced cell death with a significant reduction of total live cell numbers in femora at all three time points. Although 17-DMAG did not significantly improve the bone marrow cell viability at day 3 compared to irradiated vehicle-treated animals, 17-DMAG did markedly promote bone marrow cell viability at day 7 (17-DMAG/RI vs. vehicle/RI: 6.36 ± 1.75 × 10^5^ vs. 2.0 × 10^5^ ± 2.48 × 10^4^) and day 15 (8.26 ± 1.84 × 10^5^ vs. 3.46 × 10^5^ ± 2.91 × 10^4^) after irradiation (Figure 5A). Using a flow cytometric assay, it was determined that 17-DMAG increased bone marrow cell survival with a greater percentage of 7-aminoactinomycin D (7AAD, a death marker of cells) negative cells (Figure 5B left); their average 7AAD-negative percentages in two groups of 17-DMAG/RI and Veh/RI mice (Figure 5B right) were 72.77 ± 7.17% and 44.2 ± 4.94% (p < 0.01, n = 3), respectively, at day 15 after irradiation. The consistent results from both methods indicate that 17-DMAG protects bone marrow cells from irradiation, suggesting the improved bone marrow cell viability may contribute to increased 30-day survival in irradiated mice pretreated with 17-DMAG.

### 17-DMAG attenuated radiation-induced reduction of CD34 and CD44 expression in bone marrow cells

CD34 and CD44 are expressed in bone marrow cells. To further verify the detrimental effects of irradiation on bone marrow cells, CD34 and CD44 were measured using Western blotting 30 days postirradiation. We found that irradiation reduced the expression of both CD34 and CD44, which was prevented by 17-DMAG pre-treatment (Figure 5C). Although CD34 and 44 are not specific markers, both are usually expressed in/on hematopoietic, mesenchymal stem cells, and other types of cells in bone marrow. The improved expression of CD34 and 44 after irradiation by 17-DMAG treatment may reflect recovery of total bone marrow cells. Those data reinforced the idea that 17-DMAG protects bone marrow cells 30 days postirradiation.

### 17-DMAG increased survivin production in bone marrow cells after irradiation

Survivin is one of several anti-apoptotic proteins. It is over-produced in tumor cells and fetal tissue. Survivin is, however, expressed in normal proliferating adult cells, including human hematopoietic stem cells, T-lymphocytes, and erythroid cells throughout their maturation. It manifests its anti-apoptotic effects by inhibiting various caspases and also uniquely promotes cell mitosis and proliferation [22-24]. Inducible deletion of survivin leads to the bone marrow ablation with widespread loss of hematopoietic progenitors and rapid mortality [23].

**Figure 6:**
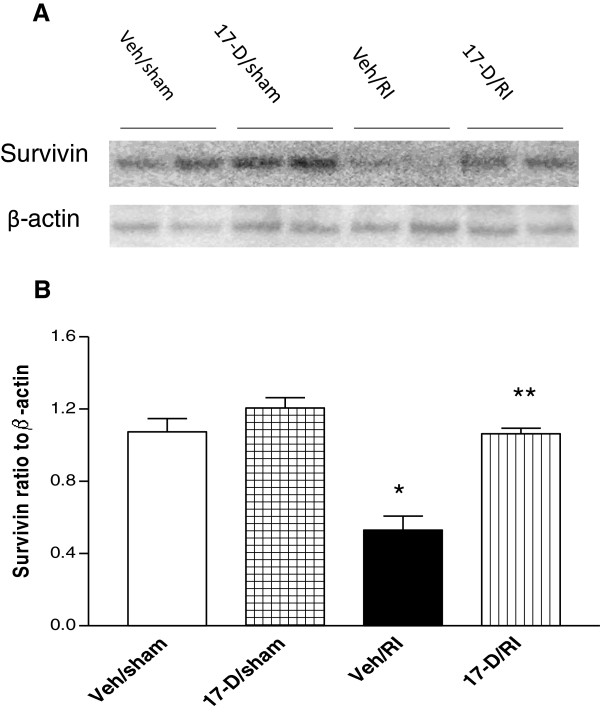
**17-DMAG increased survivin protein in bone marrow cells after irradiation.** Bone marrow cells were collected from femur bones and analyzed using Western blot for its survivin expression 30 days after irradiation at 8.75 Gy. **(A)** A representative Western blot of survivin. **(B)** Densitometric quantitation of survivin protein.* p < 0.01 vs. Veh/sham, 17-D/sham, and 17D/RI groups; ** p < 0.001 vs. Veh/RI groups. Error bars indicate the SEM for at least four independent experiments. Veh: vehicle; 17-D: 17-DMAG; RI: radiation injury.

To determine if 17-DMAG protected bone marrow cells through upregulation of survivin expression, survivin protein production in bone marrow cells of mice 30 days postirradiation was assessed using immunoblotting. Both 17-DMAG alone and 17-DMAG combined with irradiation upregulated survivin protein production in bone marrow (Figure 6), but 17-DMAG increased survivin significantly more than vehicle control at day 30 after radiation. Average survivin expression in bone marrow cells of 17-DMAG-treated irradiated animals was over two-fold of its level in bone marrow of vehicle-treated irradiated mice (Figure 6B).

### 17-DMAG upregulated serum G-CSF after irradiation

**Figure 7:**
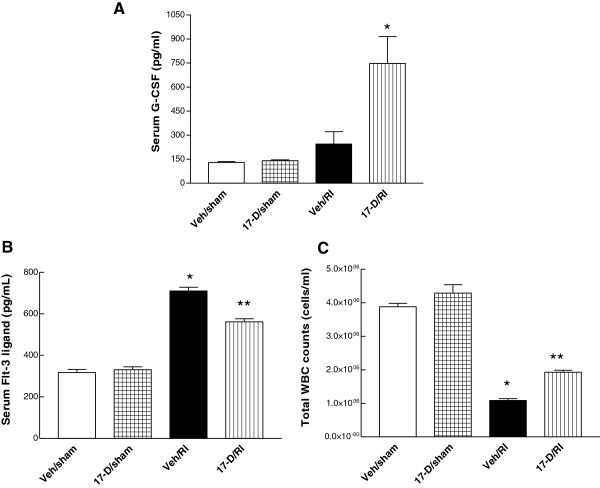
**17-DMAG upregulated serum G-CSF, inhibited radiation-induced increases in serum Flt-3 ligand, and alleviated radiation-induced WBC depletion in blood.** Mice received a single oral administration of 17-DMAG (10 mg/kg) 24 h before irradiation at 8.75 Gy. Whole blood and serum were collected 30 day after irradiation. **(A)** Serum G-CSF (pg/ml) was detected by ELISA: * p < 0.01 vs. Veh/sham, 17-D/sham, and Veh/RI groups, error bars indicating the SEM for five independent experiments. **(B)** Measurement of serum Flt-3 ligand (pg/ml) by ELISA: * p < 0.001 vs. Veh/sham, 17-D/sham, and 17-D/RI groups; ** p < 0.001 vs. Veh/sham, 17-D/sham and Veh/RI, error bars indicating the SEM for six independent experiments. **(C)** WBC counts (cells/ml): * p < 0.001 vs. Veh/sham, 17-D/sham, 17-D/RI groups; ** p < 0.001 vs. Veh/sham, 17-D/sham and Veh/RI groups, error bars also indicating the SEM for six independent experiments. Veh: vehicle; 17-D: 17-DMAG; RI: radiation injury.

Because G-CSF stimulated growth of hematopoietic stem cells in bone marrow [25], G-CSF in serum was assessed by enzyme-linked immunosorbent assay (ELISA) and immunoblotting analysis 30 days postirradiation. Irradiation (8.75 Gy) increased yet not significantly G-CSF concentration in serum, as compared to vehicle-treated sham-operated groups. 17-DMAG (10 mg/kg at −24 h), however, markedly enhanced the radiation-induced G-CSF in animal serum at day 30 after irradiation. The mean concentration of G-CSF in the irradiated animals pre-treated with 17-DMAG vs. vehicle irradiated group was 747.83 ± 186.88 vs. 244.35 ± 80.40 pg/ml, (p < 0.01, Figure 7A). Western blot data (data not shown) confirmed the observation obtained using the ELISA method as well.

### 17-DMAG inhibited radiation-induced increase in Flt-3 ligand in serum and reversed radiation-induced white blood cell (WBC) depletion in blood

Flt-3 ligand is a bio-indicator for bone marrow aplasia and its release may be triggered by stem cell deficiency in the bone marrow. Its concentration in serum is inversely correlated to the bone marrow integrity [26,27].

In our study, Flt-3 ligand concentration was measured in serum 30 days postirradiation. Irradiation notably increased Flt-3 ligand concentrations (710.02 ± 36.94 pg/ml) in serum of irradiated mice treated with vehicle (Figure 7B); whereas 17-DMAG significantly attenuated radiation-induced increase in Flt-3 ligand concentrations (548.82 ± 28.83 pg/ml, p < 0.05) in serum. Administration of 17-DMAG alone to sham-treated mice did not alter the baseline of Flt-3 concentration in serum.

To determine the radioprotective effect of 17-DMAG on circulating blood cells, peripheral blood was collected and total WBCs were counted manually at day 30. Although radiation reduced numbers of WBCs (in 10^6^ cells/ml: 1.09 ± 0.05 and 1.93 ± 0.06 of vehicle- and 17-DMAG-pretreated irradiated samples vs. 3.88 ± 0.10 and 4.23 ± 0.23 of both sham controls), 17-DMAG pretreatment increased the number of WBC 1.77 times greater (p < 0.001) than the vehicle pretreatment after irradiation (Figure 7C).

### 17-DMAG had no significant effect on radiation-induced erythropoietin (EPO) increase in serum

Because EPO produced by kidney and spleen is important for bone marrow cells [28], EPO in serum was measured 30 days after irradiation. Irradiation induced an increased EPO concentration in serum compared to non-irradiated mice (Veh + IR vs. Veh + sham: 175.84 ± 51.60 vs. 35.80 ± 7.40 pg/ml, p < 0.015). However, 17-DMAG pre-administration did not alter the radiation-induced increase in EPO concentrations compared to irradiated mice pretreated with vehicle (data not shown).

### 17-DMAG ameliorated intestinal injury induced by irradiation

**Figure 8:**
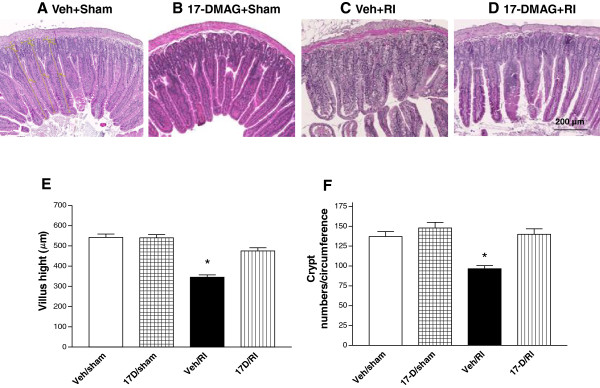
**17-DMAG ameliorated intestinal injury induced by irradiation.** Mice received a single oral administration of 17-DMAG (10 mg/kg) 24 h before irradiation at 8.75 Gy. Intestinal tissues were collected 30 days after irradiation for histological assessment. Villus morphology and height were assessed by H & E staining and observation under microscopy (10 ×) in jejunum of mice with **(A)** vehicle + sham, **(B)** 17-DMAG + sham, **(C)** vehicle + RI, and **(D)** 17-DMAG + RI at day 30 after irradiation, n = 5–6 mice per group. A 200 μm scale bar was shown on the bottom of right panel D. Occurrence of villus edema, decrease in villus heights, and ruptured villus tips were still present 30 days after the insult. 17-DMAG significantly improved recovery of intestinal histological damage with **(E)** increased villus heights (* p < 0.002 vs. 17-D/RI, error bars indicating the SEM for five or six independent experiments.) and **(F)** increased crypt numbers (* p < 0.01 vs. 17-D/RI, error bars indicating the SEM for five or six independent experiments) in transverse sections of the full jejunal circumference in 17-D/ RI mice 30 days after irradiation. Veh: vehicle; 17-D: 17-DMAG; RI: radiation injury.

To test the protective effect of 17-DMAG against radiation-induced damage of the small intestine by pre-treatment with this drug, histological analysis of the jejunum was performed 30 days post-irradiation. Small intestine of non-irradiated mice showed a histological structure of the villus intestinalis and crypts of Lieberkűhn with normal length of villus and healthy crypt cells. In contrast, irradiation induced severe pathological degeneration, which was still observed even 30 days after irradiation. A clear mucosal atrophy, denuded tips of villi were found (Figure 8C vs. A). In addition, decreased villus heights [29] and crypt numbers [30] in the circumference of intestine were observed at day 30 in surviving irradiated mice with vehicle pretreatment (Figure 8E and F), whereas 17-DMAG pre-treatment markedly improved the radiation-induced degenerative changes in the small intestine. Increased villus lengths (475.00 ± 15.99 vs. 337.53 ± 12.12 μm, p < 0.005), crypt numbers per circumference (142.95 ± 12.79 vs. 102.00 ± 6.52, p < 0.0002), and recovered small intestinal morphology in the irradiated mice pre-treated with 17-DMAG significantly differed from vehicle-control mice (Figure 8).

### 17-DMAG increased small intestinal Lgr5 expression, especially in crypt cells of irradiated mice

**Figure 9:**
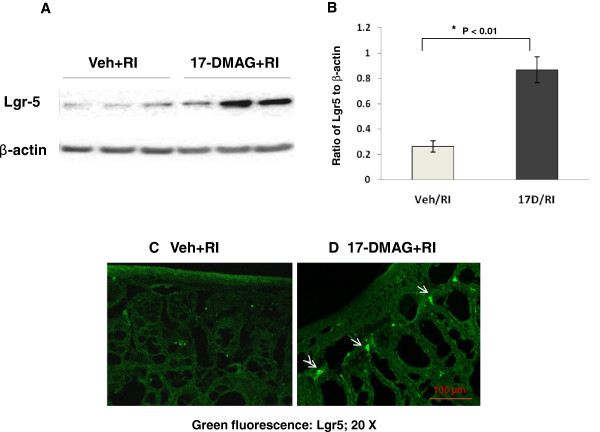
**17-DMAG upreguateded Lgr5 expression in crypt cells. (A)** Lgr5 was detected with Western blot and its expression increased in intestines of 17-DMAG-treated mice compared to vehicle-treated animals 30 days after irradiation at 8.75-Gy. **(B)** Densitometric quantitation of Lgr5 protein.* p < 0.01 vs. Veh/RI group, error bars indicating the SEM for three or four independent experiments. Lgr5 (indicated by white arrows) localizing at base cells of crypts in vehicle-pretreated **(C)** and 17-DMAG-pretreatd mice **(D)** was demonstrated using immune-staining method and confocal microscope (20 ×) 30 days after irradiation and a 100 μm scale bar was shown on the bottom. N = 3–4 mice per group.

In order to verify whether 17-DMAG has protective effect on small intestinal stem cells (ISC), Lgr5 expression in ISC was measured. Lgr5 was identified as one of markers of ISC. Lgr5-positive intestinal stem cells are crypt base columnar (CBC) cells [31] that intersperse between Paneth cells and express throughout the crypts of the intestine. Irradiation at a high dose induced ISC damage or death with a dramatic ablated Lgr5, which was observed 2 days after irradiation in murine crypt cells [32]. Herein, irradiation resulted in a decrease in the Lgr5 expression in small intestine even at day 30 after irradiation. In comparison, 17-DMAG pretreatment enhanced intestinal Lgr5 level in irradiated mice using western blot analysis (Figure 9A). The density of Lgr5 in jejunum of 17-DMAG pretreated irradiated mice was 3.34 fold (0.87 ± 0.18 vs. 0.26 ± 0.08, p < 0.01) of that in vehicle-pretreated irradiated mice (Figure 9B). Furthermore, the increased Lgr5 was located at the base of the crypt cells using immunostaining and confocal laser microscope (Figure 9C), indicating the presence of more Lgr5-positive ISC in crypts.

## Discussion

We investigated whether 17-DMAG attenuated radiation injury in bone marrow cells and the small intestine, including ISC. Although previous studies demonstrated significantly increased radiation sensitivity of tumor cells *in vivo*[4] and *in vitro*[33] in the presence of 17-DMAG, the radiosensitivity of normal fibroblasts was not altered by Hsp90 inhibition [34], suggesting that the radiosensitization induced by 17-DMAG is tumor-specific. Therefore, 17-DMAG could sensitize tumors while protecting normal tissues from radiation-induced injury or ischemia-induced infarction [34-36]. Our recent studies [13] support the idea that 17-DMAG provides protection for normal human peripheral blood mononuclear cells from irradiation.

In the present study, orally administered 17-DMAG was tested for radioprotection in CD2F1 mice. The results showed that orally administered 17-DMAG as a single dose of 10 mg/kg 24 h prior to ^60^Co γ-photon irradiation increased mouse survival by 20–25 percentage points more than corresponding controls. The radioprotective effect of orally administered 17-DMAG is only prophylactic and protective efficacy is not dose-dependent. Most importantly, the given 10 mg/kg of 17-DMAG delayed the onset of mortality by 2.5 days and significantly prolonged ST_50_ by approximately 8 days, in comparison with vehicle control after irradiation. Meanwhile, given doses of 5, 25, and 75 mg/kg were not effective, suggesting that the oral administration of 17-DMAG may have a narrow protective window. It is not clear why the repeated dose of 10 mg/kg administered to 2–3 times did not further increase radioprotective efficacy in comparison of a single dose. The effective dose, 10 mg/kg used in this study, is in agreement with its administration in the mouse model of hemorrhagic shock [37], in which orally administered 17-DMAG at a single dose of 10 mg/kg reached the maximal effect, whereas doses of 20, and 30 mg/kg did not further increase the efficacy of preventing hemorrhagic injury.

In this study, non-irradiated mice can tolerate an oral dose of 17-DMAG up to 75 mg/kg without causing any mortality 30 days after administration. This observation is in agreement with that reported by Egorin [8]. However, in irradiated mice, the optimal dose of orally administered 17-DMAG was 10 mg/kg. It is unclear why increased oral doses to 25 or 75 mg/kg failed to improve 30-day survival of irradiated animals. The oral doses of 25 and 75 mg/kg (equal to 69.67 and 209.01 mg/m^2^/day in a mouse with 25 g of body weight, respectively) exceeded the maximum tolerated dose (i.e., 16.1 mg/kg/day or 45 mg/m^2^/day) of 17-DMAG in mice [38]. Therefore, 17-DMAG at these doses might lead to some potential tissue toxicity, especially hepatotoxicity, liver dysfunction, and body weight loss [39]. We also observed body weight loss in mice pre-treated with 17-DMAG at higher doses other than those administered with 10 mg/kg (i.e., 27.87 mg/m^2^/day) from day 5 to day 25 after irradiation (data not shown). Other laboratory reported the selected nontoxic doses of oral 17-DMAG at 7.5–15 mg/kg/day (i.e., 20.9–41.8 mg/m^2^/day) were employed for an antitumor study in a carcinoma mouse model [40]. Thus, it may explain why the orally administered 17-DMAG at both high doses (may be toxic to irradiated mice) was not radioprotective. We postulate that a dose–response effect of 17-DMAG at doses less than the maximum tolerated dose could still be feasible. We also postulate that pre-administration of an oral dose of 10 mg/kg for 5 consecutive days could result in 17-DMAG accumulating in liver, because it persists in this organ for 24 h after administration [8]. In liver and kidney tissues, 17-DMAG concentration was 10-40-fold greater than it in plasma 10–1440 min after i.v. administration in mice [41]. Therefore, the accumulated drug and its degraded products day by day in liver for 5 days might add even more harm to hepatocytes and their functions that have already been impaired by irradiation.

Orally administered 17-DMAG, however, produced a lower survival efficacy than s.c. injected 17-DMAG (56% oral vs. 95% s.c. [20]) 30 days after irradiation. This discrepancy could be due to several factors. Firstly, the route of drug administration and the vehicle used were different. The optimal dose (10 mg/kg) for oral administration differed from the dose (25 mg/kg) given through s.c. injections. The vehicle used for orally administered 17-DMAG was 5% dextrose, whereas the vehicle used for s.c. injected 17-DMAG was polyethylene glycol 400 (PEG-400). Secondly, 17-DMAG bioavailability was 100% by i.v., but only 50% by oral delivery in CD2F1 mice [8] so that the drug bioavailability would be expected to be higher by a s.c. injection than by an oral delivery. Thirdly, although 17-DMAG was widely distributed to all tissues, but the highest concentration of 17-DMAG was found in the liver [8]. Orally administered 17-DMAG was initially and primarily absorbed by the gastrointestinal tract, and quickly went into liver and was much accumulated there, thereby limiting17-DMAG entering other tissues and organs. While s.c. injected 17-DMAG in PEG 400 that increased the particle size was absorbed by subcutaneous tissues and local vascular beds, and delivered to various organs through circulation without prior drug accumulation in liver. Subcutaneous tissues such as fat, in fact, can store the drug that would be released gradually because there is limited blood flow. Despite given 25 mg/kg of 17-DMAG in PEG-400 via s.c. injection, being higher than maximum tolerated dose per day, it was generally absorbed more slowly, even taking more than 24–48 h into blood. Thus, a greater radioprotective efficacy of s.c. injected 17-DMAG than orally administered 17-DMAG was observed.

We postulate that most of the orally administered 17-DMAG at 25 or 75 mg/kg might have been accumulated, metabolized, and degraded in the livers of sham animals. These processes would be critical to eliminate the drug and its degraded products from the body, otherwise producing potential harmful side effects or tissue toxicity in 48–72 h. In irradiated mice, the livers were incapable or limitable of performing detoxification due to impairment caused from the lethal whole-body irradiation. As a result of this failure to detoxify, the pharmacologic effects of 17-DMAG at high doses decrease or become detrimental. Only a nontoxic oral dose of 17-DMAG at 10 mg/kg enable to retain radioprotection.

We noticed that repeated 17-DMAG administrations at 10 mg/kg failed to further improve the mouse survival after irradiation. We hypothesize that it is possible due to degraded products of 17-DMAG accumulating in an impaired liver caused by irradiation. This accumulation of degraded products resulted in toxicity to the irradiated mice. The same explanation may also explain why orally administered 17-DMAG for 5 consecutive days before irradiation failed to improve the mouse survival after irradiation.

Post-treatment with 17-DMAG also failed to improve survival after irradiation, due to radiation-induced DNA double strand breaks (DSB) and p53 phosphorylation, which occurred within 1 h after irradiation. Those events were then followed by sequential activations of signal transduction pathways leading to cell death and multiple organ dysfunction and failure. We have reported that pretreatment of human T cells with 17-DMAG was effective in preventing DSB and p53 phosphorylation by blocking p53 interaction with Hsp 90, and p53 gene knockout cells were also resistant to irradiation [13]. This likely explains why in this presented study pretreatment with 17-DMAG is effective. Post-treatment with 17-DMAG probably would be too late to stop DSB and p53 phosphorylation, which would explain why the post-treatment with the drug is ineffective.

Radiation significantly reduced body weight and water consumption in CD2F1 mice (Figure 3); these observations agree with those in B6D2F1/J mice [21]. Radiation-induced facial dropsy was found in CD2F1 mice [42], C57BL/6 mice, and iNOS knockout mice, but not B6D2F1/J mice (data not shown), suggesting this observation could be strain-specific. It is highly likely that 17-DMAG inhibition on radiation-induced facial edema may involve in its activity of anti-inflammation, but more experiments will be necessary.

Radiation significantly depleted bone marrow cells and induced adipose vacuole appearance (Figure 4C). In 17-DMAG-treated mice, amelioration of bone marrow cell depletion, accelerated recovery of the cellularity, and reduced adipogenesis of bone marrow (Figure 4D) were observed accompanied by upregulation of CD34 and CD44 expression (Figure 5C), indicating that 17-DMAG could protect bone marrow cells of 30-day surviving mice after irradiation. The view is further reinforced by increased numbers of live bone marrow cells at days 7 and 15 and elevated 7AAD-negative percentage of bone marrow cells at day 15. However, how 17-DMAG exerting its radioprotection on bone marrow cells remains unknown.

Survivin is normally bound to Hsp90 in the cytoplasm [22]. It is reported that cultured SW480 cell exposed to a 4-Gy irradiation [43], the nuclear survivin was linked to DNA double-strand-break repair by interaction with members of the repair pathway. This view is supported by a study using normal healthy human peripheral blood mononuclear cells, in which 17-DMAG inhibited radiation-induced increases in γ-H2AX, a biomarker of DSB [13]. Although irradiation increased the survivin protein level at day 30 after irradiation, we think this increase is a self-defensive response which is too late to alleviate the mortality. We postulate that 17-DMAG inhibits Hsp90 resulting in the release of survivin from Hsp90 and then survivin ready for involving in the process of repairing the radiation-induced DSB.

Our survivin data agree with findings from another laboratory [44], in which 17-DMAG (0.1–1 μg/ml) alone elevated survivin production *in vitro* and rendered the cells insensitive to apoptosis. Survivin is anti-apoptotic by inhibiting various caspases [22,24]. Our laboratory reported that 17-DMAG inhibited caspase-3 and -9, leading to an inhibition of apoptosis [12,37]. It is plausible that 17-DMAG increased mouse survival after irradiation by activating the survivin pathway that may regulate β-catenin, p53, NF-κB, and Stat3 multiple signalings [45] and caspase-3 and -7 [24]. Further investigation of survivin regulation by 17-DMAG is warranted.

Moreover, the 17-DMAG ameliorating bone marrow damage induced by irradiation correlates with the reduction of Flt-3 ligand concentrations in serum (Figure 7B). The serum level of Flt-3 ligand has been suggested as a biomarker of radiation injury to bone marrow and a surrogate for the extent of damage to hematopoietic progenitor cells in bone marrow after ionizing irradiation [46-48]. Thus, the reduction of radiation-induced higher Flt-3 ligand concentrations is another indirect evidence to support the radioprotection of bone marrow cells by 17-DMAG. Our result on Flt-3 ligand concentrations is consistent with that in another report [46] that the concentration of serum Flt-3 ligand is reversely correlated with WBC counts in peripheral blood.

17-DMAG attenuated the radiation-induced WBC depletion (Figure 7C), which correlated with G-CSF increases in the presence of 17-DMAG (Figure 7A). G-CSF acts at all stages of neutrophil development, specifically increasing the proliferation and differentiation of neutrophils from committed progenitors [49], enhancing survival and function of mature neutrophils [50,51]. G-CSF is a cytokine and a growth factor, which possesses radioprotective properties [52,53] by stimulating growth, differentiation and prevention of apoptosis of progenitor cells. Like 17-DMAG, pre-administration of alpha-tocopherol succinate [54,55] and post-treatment with meloxicam [56] or 5-androstenediol [57] increased serum G-CSF concentrations within 4–24 h after injections. Alpha-tocopherol succinate induced a peak level of G-CSF within 24 h and a rapid fall 36 h after s.c. administration. Our other data from irradiated CD2F1 mice pre-treated s.c. with 17-DMAG also indicated that 17-DMAG enhanced serum G-CSF concentration as early as 4 h and lasted up to day 2, 10, and 15 after irradiation compared with the vehicle group (Lu et al., unpublished data). These data suggest that 17-DMAG, like other radioprotectant such as alpha-tocopherol succinate, confers radioprotection by inducing high levels of G-CSF. Other studies indicated that G-CSF not only enhanced the production of hematopoietic progenitor cells in bone marrow but also mobilized those primitive progenitors from the hematopoietic tissue into the circulation [58,59]. Therefore, 17-DMAG administration increased numbers of WBC probably mediated by the increasing G-CSF 30 days postirradiation. The present study may offer a new insight on regulation of survivin and G-CSF by 17-DMAG. However, the possibility of increased G-CSF concentrations being a consequence of hematopoietic recovery cannot be excluded.

It is known that 17-DMAG inhibits activation of the iNOS pathway *in vitro*[37] and *in vivo*[20], and the p53 pathway *ex vivo*[13]. Further studies should explore the inter-relationship among iNOS, p53, and survivin pathways and the regulation of G-CSF.

Radiation-induced gastrointestinal syndrome (RIGS) results from a combination of direct cytocidal effects on intestinal crypt and endothelial cells and subsequent loss of the mucosal barrier, leading to malabsorption, electrolyte imbalance, diarrhea, weight loss, infection, dysfunction, and final mortality. Stem cells located at the base of the crypt undergo rapid apoptosis or stop dividing temporarily or permanently after irradiation. Therefore, RIGS is due in part to the killing of clonogenic crypt cells with eventual depopulation of the intestinal villi [60].

17-DMAG attenuated the pathological alteration in villi 30 days postirradiation. Oral administration of 17-DMAG (10 mg/kg) decreased mucosal atrophy, edema and ulceration, increased the number of crypts where ISC are located, and more importantly, upregulated expression of Lgr5 (a molecular marker of ISC in intestinal crypts). The results agree with the findings in hemorrhaged jejunum [37], suggesting that 17-DMAG prevents the radiation-induced structural injury in small intestine, body weight loss, and facial edema, further improves survival of irradiated mice.

Since in hemorrhaged small intestine, 17-DMAG diminished hemorrhage-induced small intestine injury by elevating Bcl-2 protein and inhibiting iNOS pathway, TNF-α production, and caspase-3 activation [37], the possibility of 17-DMAG exerting its actions on these factors to ameliorate the radiation-induced gastrointestinal injury cannot be excluded. More studies in this regard are ongoing.

## Conclusion

A single dose of orally administered 17-DMAG (10 mg/kg) as a prophylactic measure prior to irradiation was effective to increase mouse 30-day survival by protecting bone marrow and small intestine from radiation injury. The protective effects of pre-administered 17-DMAG were associated with (1) increased expression of survivin, CD34, CD44 in bone marrow and G-CSF in serum, which were correlated with attenuated bone marrow injury and WBC loss and were confirmed by the decreased Flt-3 ligand concentration in circulation; (2) up-regulated Lgr5 expression and Lgr5-positive stem cells in intestinal crypts, which promoted intestinal epithelial repairing, self-renewing and body weight recovery from irradiation-induced injury. Taken together, our results suggest that 17-DMAG appears to be an effective prophylactic radioprotectant in bone marrow cells and in intestinal crypt cells including intestinal stem cells, which contributes to enhanced survival.

## Materials and methods

### Animals

Male CD2F1 mice were purchased from Harlan Laboratories (Dublin, VA, USA) and used at age of 10 to 12 weeks at the time of irradiation. The mice were housed in groups of four in polycarbonate microisolator cages (11.5 × 7.5 × 5 in.) with filter tops on autoclaved rodent hardwood bedding. Animal rooms were maintained at 21 ± 2°C, 50% ± 10% humidity, and 12-h light/dark cycle. All facilities were accredited by the Association for Assessment and Accreditation of Laboratory Animal Care International. Rodent food (Harlan Teklad Rodent Diet 8604) and water were freely available for mice. All handling procedures were performed in compliance with guidelines from the National Research Council, and were approved by the Institutional Animal Care and Use Committee (IACUC) of the AFRRI.

### 17-DMAG preparation and oral administration

17-DMAG (LC Laboratories, Woburn, MA, USA) was prepared as previously described [37]. Briefly, 17-DMAG was freshly solubilized in 5% Dextrose (Baxter Healthcare, Deerfield, IL, USA) on the day of the experiment. The drug was orally administered to mice at (1) a single dose of 5, 10, 25, or 75 mg/kg in a volume of 0.2 ml 24 h before irradiation; (2) a single dose of 10 mg/kg 1, 24, 48, or 72 h prior to irradiation; (3) a single dose of 10 mg/kg 1, 6, or 24 h after irradiation; and (4) multiple doses of 10 mg/kg 120, 96, 72, 48 and 24 h before irradiation. Vehicle groups were orally fed the same volume of 5% Dextrose.

### Irradiation

Mice were restrained in well-ventilated acrylic irradiation boxes. Mice were given a dose of 8.75 Gy ^60^Co γ photons at a dose rate of 0.6 Gy/min bilaterally in the AFRRI ^60^Co Irradiation Facility. The uniformity of the field in the used part of the rack was ± 1%. Control (sham) animals were placed into the acrylic radiation boxes but were not irradiated. The total tissue dose of radiation received was measured at the level of the abdominal core. An alanine electron-spin-resonance (ESR) dosimetry system (American Society for Testing and Materials, Standard E 1607) was used to measure dose rates (to water) in the cores of acrylic mouse phantoms. To simulate a mouse, the phantoms were three inches in length and one inch in diameter. For field mapping, all exposure rack compartments contained phantoms, and alternate phantoms contained alanine dosimeters. The ESR signals were measured using a calibration curve based on the standard calibration dosimeters (National Institute of Standards and Technology (NIST), Gaithersburg, MD, USA). The overall uncertainty in the doses given to the calibration dosimeters at NIST was approximately 1.8% at 2 standard deviations. The accuracy of the calibration curve was verified by parallel measurements of doses to selected dosimeters at AFRRI and the National Physical Laboratory, Middlesex, England, UK. Corrections were applied to the dose rates in phantoms for the decay of ^60^Co and differences in the mass energy-absorption coefficients for water and soft tissue [61]. The day of irradiation was considered day 0.

### Animal survival, body weight, water consumption, and facial dropsy

Sixteen mice were randomly assigned to each group. Experimental groups included: (1) 5% Dextrose vehicle + sham (Veh/sham), (2) 17-DMAG + sham (17-D/sham), (3) 5% Dextrose vehicle + radiation injury (Veh/RI), and (4) 17-DMAG + radiation injury (17-D/RI). After irradiation, mice were returned to their home cages. Water consumption was measured with graduated water bottles daily for the first 7 days. Mouse survival, body weight, and facial dropsy were monitored for 30 days post-irradiation. At day 30, the surviving mice were euthanized and their bloods, intestines and femurs were collected for analysis.

### Histological examination, bone marrow myeloid cell viability, and cell phenotype analysis

One femur from each surviving mouse 30 days after irradiation was fixed in 10% formalin for at least 24 h. All samples were embedded with paraffin and sectioned longitudinally for haematoxylin & eosin (H & E) staining. The stained slides were examined at 20 × magnification under microscope (Olympus BX-61, Olympus, Japan). Bone marrow cells were flushed from the other femur of each surviving mouse 30 days after irradiation. After two washes with phosphate buffered saline (PBS) solution, cells were lysed by using lysis buffer and their proteins were extracted following a commercial extraction method (Pierce Protein Research, Rockford, IL, USA). For investigating effect of 17-DMAG on bone marrow after irradiation, bone marrow cells were also collected from mouse femora at 3, 7 and 15 days after irradiation in a separate experiment. After erythrocytes were lysed by red blood cell lysis buffer (BioLegend San Diego, CA, USA), total bone marrow cell viability from each mouse was assayed by trypan blue staining [12]. For bone marrow cells from the mice at day 15 after irradiation, cells also were labeled with 7AAD, a death marker of cells and their cell viabilities were analyzed by flow cytometry using BD FACSCalibur (BD Biosciences, San Jose, CA, USA). Cells were gated for 7AAD-positive cells and negative live cells. All antibodies and dyes were purchased from BD Biosciences.

### Intestinal histological and immunohistochemical assessments

Small intestine was harvested after 30-day survival for intestinal protein expression or morphological analysis. The distal jejunum and proximal ileum were fixed for histology in 10% formalin with PBS. Paraffin embedded gut tissues were sectioned and stained with H & E. Nanozoomer virtual microscopy (Hamamatsu Photonics, Hamamatsu, Japan) was used for measurement of the villus height and the count of crypt numbers. A minimum of 10 well-oriented villi per tissue section (at least 5 sections from each gut specimen) was measured and crypt numbers were counted in all 5 sections of each specimen.

Frozen slides of ileum were prepared and immunostained, following a previous published method [62] for expression and localization of Lgr5 in small intestine. Briefly, after fixing and blocking procedure, slides were incubated with primary antibodies, goat anti-Lgr5 IgG (Genway, SanDiego, CA, USA) 1:100 dilution for 60 min. Slides were incubated with secondary antibodies, donkey anti-goat IgG-Alexa Fluor 488 (Invitrogen, Carlsbad, CA, USA) for 1 h. Finally, slides were mounted with ProLong Gold Antifade, counterstained with DAPI (4’ ,6’-diamidino-2-phenylindole; Invitrogen, Carlsbad, CA, USA), and observed under a confocal laser scanning microscope (Zeiss LSM710, Carl Zeiss MicroImaging, Thornwood, NY, USA).

### Western blot

Proteins in lysates of bone marrow and gut tissues were separated by 4-12% Bis-tris gel electrophoresis and transferred onto a nitrocellulose membrane according to a standard technique for antibody detection of proteins. Primary antibodies against mouse G-CSF (BD Pharmingen, San Diego, CA, USA), CD44 (BioLegend, San Diego, CA, USA), CD34 (Santa Cruz Biotechnology, Santa Cruz, CA, USA), Lgr5 (Genway, SanDiego, CA, USA), survivin (Millipore, Temecula, CA, USA), and β-actin (Sigma, St. Louis, MO, USA) were used in immunoblotting assays, respectively.

### Enzyme-linked immunosorbent assay

Murine serum levels of EPO, G-CSF, and Flt-3 ligand were measured by ELISA (R&D Systems, Minneapolis, MN, USA) according to the manufacturer’s protocols.

### White blood cell counts

Blood was collected by cardiac puncture from mice anesthetized with isoflurane inhalation and placed promptly in an ethylenediaminetetraacetic acid (EDTA)-containing tube for blood cell counts and a regular eppendorf tube for serum separation. Red blood cells in the EDTA-containing tube were lysed by RBC lysis buffer (BioLegend San Diego, CA, USA), and the remaining leukocytes were washed in 1% ammonium oxalate solution. The total number of leukocytes in 10 μl of blood was counted manually in a hemocytometer.

### Statistical analysis

The data of 30-day survival of mice were analyzed using Two-way analysis of variance (ANOVA). For parametric data, differences among or between groups were compared by ANOVA and Student’s *t*-test, respectively, with significance at p < 0.05. Results of parametric data were presented as means ± standard errors.

## Abbreviations

7AAD: 7-aminoactinomycin D; 17-DMAG: 17-dimethylamino-ethylamino-17-demethoxygeldanamycin; CBC: Crypt base columnar; CD34, CD44: Cluster of differentiation 34, 44; DMF: Dose-modifying factor; DRF: Dose reduction factor; DSB: DNA double strand breaks; EPO: Erythropoietin; Flt-3: fms-like tyrosine kinase-3; G-CSF: Granulocyte-colony stimulating factor; Hsp90: Heat shock protein 90; iNOS: Inducible nitric oxide synthase; ISC: Intestinal stem cells; i.v.: Intravenous; LD70/30: A dose to result in 70 percent of an exposed population died within 30 days; Lgr5: Leucine-rich repeat-containing G-protein coupled receptor 5; RBC: Red blood cell; s.c.: Subcutaneous; Veh/sham: Vehicle + sham; ST50: Median survival time; 17-D/sham: 17-DMAG + sham; Veh/RI: Vehicle + radiation injury; 17-D/RI: 17-DMAG + radiation injury; WBC: White blood cell.

## Competing interests

The authors declare that they have no competing interests. The authors alone are responsible for the content and writing of the paper. The views, opinions and findings contained in this report are those of the authors and do not reflect official policy or positions of the US Department of the Navy, the US Department of Defense, or the United States Government.

## Author’s contributions

XL carried out the experimental studies, data analysis and drafted the manuscript. DN conducted Western blot and animal experiment. DLB contributed to animal irradiation, TBE participated in animal experiment and revised the manuscript. JGK conceived the project, designed the study, reviewed and revised final manuscript. All authors read and agreed with the manuscript. The funders had no role in study design, data collection and analysis or preparation of the manuscript.
